# Delivering Medical Abortion at Scale: A Study of the Retail Market for Medical Abortion in Madhya Pradesh, India

**DOI:** 10.1371/journal.pone.0120637

**Published:** 2015-03-30

**Authors:** Timothy Powell-Jackson, Rajib Acharya, Veronique Filippi, Carine Ronsmans

**Affiliations:** 1 Department of Global Health and Development, Faculty of Public Health and Policy, London School Hygiene and Tropical Medicine, London, United Kingdom; 2 Population Council, New Delhi, India; 3 Department of Infectious Disease Epidemiology, Faculty of Epidemiology and Population Health, London School Hygiene and Tropical Medicine, London, United Kingdom; NHS lothian and University of Edinburgh, UNITED KINGDOM

## Abstract

**Background:**

Medical abortion (mifepristone and misoprostol) has the potential to contribute to reduced maternal mortality but little is known about the provision or quality of advice for medical abortion through the private retail sector. We examined the availability of medical abortion and the practices of pharmacists in India, where abortion has been legal since 1972.

**Methods:**

We interviewed 591 pharmacists in 60 local markets in city, town and rural areas of Madhya Pradesh. One month later, we returned to 359 pharmacists with undercover patients who presented themselves unannounced as genuine customers seeking a medical abortion.

**Results:**

Medical abortion was offered to undercover patients by 256 (71.3%) pharmacists and 24 different brands were identified. Two thirds (68.5%) of pharmacists stated that abortion was illegal in India. Only 106 (38.5%) pharmacists asked clients the timing of the last menstrual period and 38 (13.8%) requested to see a doctor’s prescription – a legal requirement in India. Only 59 (21.5%) pharmacists correctly advised patients on the gestational limit for medical abortion, 97 (35.3%) provided correct information on how many and when to take the tablets in a combination pack, and 78 (28.4%) gave accurate advice on where to seek care in case of complications. Advice on post-abortion family planning was almost nonexistent.

**Conclusions:**

The retail market for medical abortion is extensive, but the quality of advice given to patients is poor. Although the contribution of medical abortion to women’s health in India is poorly understood, there is an urgent need to improve the practices of pharmacists selling medical abortion.

## Background

The proportion of abortions that are unsafe has increased in recent years [1] and abortion continues to be a major cause of maternal death, accounting for between 7.9% and 14.9% of maternal deaths worldwide.[2, 3] Of global importance is India where one-quarter of global maternal deaths are found [2] and 8.8% of maternal deaths are due to abortion.[4]

A pivotal medical advance in the past few decades has been the development of medical abortion—the use of pills to cause an abortion.[5] Mifepristone combined with misoprostol is effective, safe and acceptable in various settings,[6–8] including India.[9–11] The private retail market for medical abortion is thought to be expanding rapidly in India, with sales of misoprostol showing a dramatic increase between 2002 and 2007.[12] However, little is known about the provision or quality of advice for medical abortion through the private retail sector.

Abortion has been legal in India since 1972, when the Medical Termination of Pregnancy Act came into effect, allowing termination of pregnancy up to 20 weeks gestation under certain broad conditions. In 2003, an amendment to the regulations permitted medical practitioners certified to provide surgical abortion to prescribe mifepristone with misoprostol for medical abortion of pregnancies up to 49 days, on condition the woman has access to a certified abortion facility if complications arise. Despite the liberal abortion law, awareness that abortion is legal is still limited and concerns about confidentiality, cost and quality of care lead women to choose uncertified providers closer to home.[13, 14] Abortion services are often not available at the primary care level in the public sector, and some facilities may lack trained staff, drugs or equipment necessary for a safe abortion.[15]

We undertook an observational study to understand the scale and quality of the retail market for medical abortion in Madhya Pradesh, India. We mapped all health providers in selected markets, interviewed private pharmacists, returning one month later with undercover patients to collect information on the availability of medical abortion, the knowledge of the person behind the counter, and the quality of advice given to customers. As efforts are made to reduce unsafe abortions, it is crucial that better evidence be made available on the role of the retail market in the provision of medical abortion.

## Methods

### Study setting

The study took place in the state of Madhya Pradesh, with a population of 73 million living in 50 districts.[16] Two thirds of the population lives in rural areas and 34% of women are illiterate. The maternal mortality ratio was estimated at 277/100,000 live births in 2011–12.[17] Current use of modern contraception is 59%, of which the vast majority is female sterilisation.[17] Only 10% of women use the pill, IUD, injections or condoms. Son preference is strong, as indicated by a male to female sex ratio at birth of 1.10 in 2011–2012.[17]

### Sample selection

All districts in Madhya Pradesh were stratified into two groups according to the proportion of the population living in urban areas in 2011.[16] We randomly selected three highly urbanised districts (Bhopal, Indore, and Gwalior) and three rural districts (Satna, Ashoknagar, and Umaria) (S1 Table). Within each district we randomly selected 10 sampling units in proportion to sub-district population size.[16] A sampling unit was either an administrative ward in a city, a block headquarters in a town, or a rural village with a population over 3,000. Smaller villages were excluded because initial research suggested we would find no pharmacists. Field researchers then identified the local market (an agglomeration of pharmacists) in each sampling unit, took a centre point and limited the area for enumeration to a 1km radius in city areas, and a 2km radius in town and rural areas. The final selection included 20 city, 25 town and 15 rural local markets.

In each local market, every healthcare provider in the public and private sector was visited and mapped by their GPS location. Pharmacies were defined as any outlet whose primary business was selling medicines, irrespective of whether they were appropriately trained as required by law. A fixed number of 15 pharmacies (or less where the local market had fewer than 15 pharmacies) were selected for interview by an equal probability systematic sampling. We returned to a random sub-sample of the interviewed pharmacists to gather data using undercover patients.

### Data collection

Trained field researchers interviewed the person behind the counter using a structured questionnaire on the pharmacist’s background characteristics, the sales, stocks and price of medical abortion, and the pharmacist’s knowledge of abortion. Information on drugs was collected for each brand of single misoprostol tablets and combination pack of mifepristone with misoprostol. Knowledge tested included the legal status of abortion in India, the gestational limit for medical abortion, and the dosage and directions of use. A vignette of a medical abortion customer was also used to examine the pharmacist’s knowledge of what questions should be asked to the customer, what advice should be given, and the warning signs of potential complications from taking the drugs. The field researchers did not prompt for answers to these questions. All interviews were done in September 2013 by 10 trained field researchers.

We observed the actual quality of advice using a mystery client methodology.[18] One month after the interview of pharmacists, undercover patients presented themselves unannounced as a customer interested in purchasing a drug without a prescription to induce an abortion. They were selected to conform closely to the typical customer seen by pharmacists in the study districts. We used three male and two female clients using two scenarios: a woman aged 25–30 years who was six weeks pregnant, had heard about medical abortion and was seeking to end the pregnancy; and a man aged 28–30 who was seeking to purchase the drugs on behalf of his wife who was six weeks pregnant. The undercover patients were trained to rehearse a standardised script through extensive role play and pre-tests, and to recall accurately the interaction with the pharmacist.

Within an hour of their visit the undercover patients met with their supervisor who used a structured questionnaire to note the advice given about dosage and directions of use of the drugs, potential side-effects, warning signs and the need to seek care, and post-abortion family planning. If no advice was volunteered by the pharmacist, the undercover patients prompted for information. No medications were purchased since the undercover patients were not carrying a doctor’s prescription; they were instead trained to offer an excuse and end the interaction.

The research ethics committees of the Futures Group in India, Population Council, and London School of Hygiene and Tropical Medicine approved the study (S1 Appendix). Pharmacists provided written informed consent to be interviewed.

### Statistical analysis

We defined medical abortion as misoprostol and mifepristone sold in combination packs or separately. Availability of medical abortion was measured in three ways: sold by the pharmacist in the past year; currently in stock at interview; and offered to the undercover patient. We examined availability at the pharmacist level and within a local market (i.e. available in at least one pharmacist). Results were stratified by city, town and rural areas and differences were examined using a χ^2^ test.

The responses to knowledge questions were coded as either correct, incorrect, or don’t know. The knowledge of pharmacists measured using the vignette of a medical abortion customer was evaluated against a checklist of recommended practice.[19] Finally, we compared practice and knowledge amongst pharmacists who offered undercover patients any drug(s) to induce an abortion for a set of indicators that were available in both datasets, using a Wilcoxon signed rank sum test. Data were analysed using Stata (version 13).

## Results

### Provision of healthcare and characteristics of pharmacists

**Fig 1:**
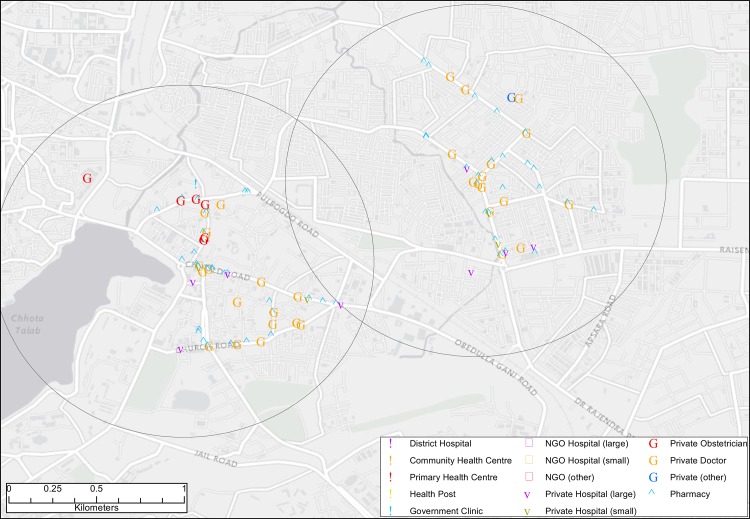
Illustration of healthcare provision in a city local market.

We mapped 1,090 pharmacies in 60 local markets (Fig. 1). The health services available in each local market are shown in Table 1. Each local market had a median of 13 pharmacies (range 0–67), 11 private doctors (range 0–57), and 1 private hospital (range 0–27), far exceeding the provision of care in the public sector. The median number of pharmacies was 32 in city (range 7–67), 13 in town (range 3–46) and 2 in rural local markets (range 0–9). Three rural markets had no pharmacies.

**Table 1:** Healthcare provision at the local market level.

	Total	City	Town (block)	Rural (village)
Number of government hospitals	0 (0–1)	0 (0–1)	0 (0–1)	0 (0–0)
Number of government community health centre	0 (0–1)	0 (0–1)	1 (0–1)	0 (0–1)
Number of government primary health centre	0 (0–1)	0 (0–1)	0 (0–1)	0 (0–1)
Number of government health post or sub health centre	0 (0–1)	0 (0–0)	0 (0–0)	0 (0–1)
Number of private hospitals	1 (0–27)	7 (1–27)	1 (0–5)	0 (0–1)
Number of private gynaecologists	0 (0–6)	0 (0–6)	0 (0–1)	0 (0–0)
Number of private doctors	11 (0–57)	19 (0–57)	9 (2–42)	4 (1–19)
Number of pharmacists	13 (0–67)	32 (7–67)	13 (3–46)	2 (0–9)

The table reports median values and the range in parentheses. There were 60 local markets, of which 3 contained no pharmacists.

Of the 680 pharmacies sampled, 591 (87%) agreed to be interviewed. The pharmacists were mostly Hindu, well educated, open every day of the week, and busy (S2 Table). Undercover patients returned to 359 pharmacists whose characteristics were similar to those interviewed (S3 Table).

### Availability of medical abortion

Table 2 shows that 187 (31.6%) pharmacists reported selling medical abortion in the past year and 132 (22.3%) had the drugs in stock when interviewed. When presented with an undercover patient, 256 (71.3%) pharmacists offered medical abortion. The market for medical abortion was dominated by combination packs. 74 (20.6%) pharmacists offered undercover patients traditional (ayurvedic) or alternative (homeopathic) drugs (data not shown). There were no significant differences by type of location.

**Table 2:** Pharmacy availability of medical abortion.

	Total		City		Town (block)		Rural (village)		P value of difference
	n	Availability	n	Availability	n	Availability	n	Availability	
**Interview data**									
Sold by pharmacist in past year	591	187 (31.6%)	283	86 (30.4%)	265	85 (32.1%)	43	16 (37.2%)	0.655
Combination pack of medical abortion	591	180 (30.5%)	283	82 (29.0%)	265	84 (31.7%)	43	14 (32.6%)	0.750
Separately sold mifepristone and misoprostol	591	20 (3.4%)	283	9 (3.2%)	265	9 (3.4%)	43	2 (4.7%)	0.884
Currently in stock	591	132 (22.3%)	283	67 (23.7%)	265	56 (21.1%)	43	9 (20.9%)	0.755
Combination pack of medical abortion	591	128 (21.7%)	283	64 (22.6%)	265	55 (20.8%)	43	9 (20.9%)	0.863
Separately sold mifepristone and misoprostol	591	10 (1.7%)	283	5 (1.8%)	265	5 (1.9%)	43	0 (0.0%)	0.667
**Undercover patient data**									
Offered to client for sale	359	256 (71.3%)	162	108 (66.7%)	162	123 (75.9%)	35	25 (71.4%)	0.183
Combination pack of medical abortion	359	241 (67.1%)	162	101 (62.4%)	162	117 (72.2%)	35	23 (65.7%)	0.164
Separately sold mifepristone and misoprostol	359	15 (4.2%)	162	7 (4.3%)	162	6 (3.7%)	35	2 (5.7%)	0.858

P value is from a χ^2^ test of the difference between the three types of location.

Availability of medical abortion in local markets was high (Table 3): 50 (83.3%) local markets had at least one pharmacist selling medical abortion in the past year and 37 (61.7%) when measured in terms of current stock. Data from the undercover patients show that medical abortion was available in 52 (86.7%) local markets. Availability was almost universal in city and town areas but substantially lower in rural areas.

**Table 3:** Local market availability of medical abortion.

	Total		City		Town (block)		Rural (village)		P value of difference
	n	Availability	n	Availability	n	Availability	n	Availability	
**Interview data**									
Sold in past year in local market	60	50 (83.3%)	20	19 (95.0%)	25	23 (92.0%)	15	8 (53.3%)	0.001
Combination pack of medical abortion	60	48 (80.0%)	20	18 (90.0%)	25	23 (92.0%)	15	7 (46.7%)	0.001
Separately sold mifepristone and misoprostol	60	17 (28.3%)	20	8 (40.0%)	25	8 (32.0%)	15	1 (6.7%)	0.083
Currently in stock in local market	60	37 (61.7%)	20	17 (85.0%)	25	15 (60.0%)	15	5 (33.3%)	0.008
Combination pack of medical abortion	60	37 (61.7%)	20	17 (85.0%)	25	15 (60.0%)	15	5 (33.3%)	0.008
Separately sold mifepristone and misoprostol	60	9 (15.0%)	20	4 (20.0%)	25	5 (20.0%)	15	0 (0.0%)	0.037
**Undercover patient data**									
Offered to client for sale in local market	60	52 (86.7%)	20	19 (95.0%)	25	25 (100.0%)	15	8 (53.3%)	<0.001
Combination pack of medical abortion	60	51 (85.0%)	20	19 (95.0%)	25	24 (96.0%)	15	8 (53.3%)	<0.001
Separately sold mifepristone and misoprostol	60	14 (23.3%)	20	7 (35.0%)	25	6 (24.0%)	15	1 (6.7%)	0.145

Medical abortion is available at the local market level if medical abortion is available in at least one pharmacist. Estimates take into account the three rural local markets that contained no pharmacists to be interviewed. P value is from a χ^2^ test of the difference between the three types of location.

The interview data suggest that 15 brands of combination pack were available in stock, while undercover patients were offered 24 different brands (S1 Fig.). The range of brands was greatest in town areas. In local markets where combination packs were available, the median number of brands was 2 or 3 depending on data source.

**Fig 2:**
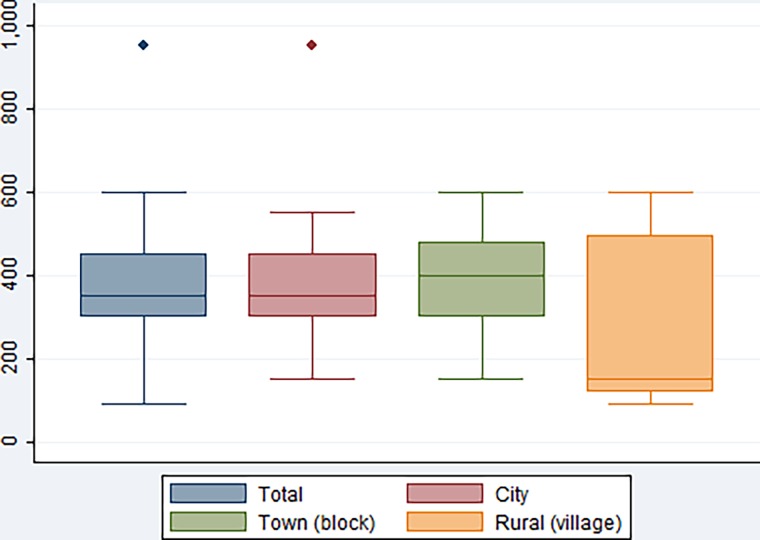
Price of medical abortion combination packs. Central line shows the median value and the box shows the interquartile range (IQR), while the whiskers represent range of prices. Circles represent statistical outliers—i e, individual chemists with prices outside the range: first quartile–(1.5×IQR) to third quartile+(1.5×IQR).

The median price of combination packs was 350 Indian Rupees (£3.82) (Fig. 2). The price was highest in town areas (median 397 Indian Rupees (£4.33)) and lowest in rural areas (median 150 Indian Rupees (£1.64)).

### Knowledge of abortion

Two thirds (68.5%) of pharmacists stated that abortion was illegal in India (Table 4). While 347 (58.7%) pharmacists correctly reported how to calculate gestational age, 199 (33.7%) did not know. One third of pharmacists (31.0%) knew the legal gestational limit for medical abortion in India. When asked the dosage and timing of drugs in combination packs, 44.8% stated that they did not know. Knowledge was similar amongst the sample of pharmacists who were approached by an undercover patient (S4 Table). Knowledge of what questions to ask, what advice should be given, and the warning signs of potential complications were probed using the data from a vignette of a medical abortion customer (S5 Table).

**Table 4:** Knowledge of abortion (interview data).

	Number of pharmacists	Knowledge
**Is abortion legal in India?**		
Correct response (yes)	591	167 (28.3%)
Incorrect response (no)	591	405 (68.5%)
“Don’t know”	591	19 (3.2%)
**How do you calculate gestation age?**		
Correct response (beginning of last menstrual period)	591	347 (58.7%)
Incorrect response (any other response)	591	45 (7.6%)
“Don’t know”	591	199 (33.7%)
**Medical abortion is permissible up to how many weeks pregnant?**		
Correct response (between 7 and 9 weeks inclusive)	591	183 (31.0%)
Incorrect (more than 9 weeks)	591	164 (27.7%)
Incorrect (less than 7 weeks)	591	163 (27.6%)
“Don’t know”	591	81 (13.7%)
**How should combination pack medical abortion drugs be administered (number of pills and timing)?**		
Correct response (one tablet of mifepristone on day one, followed by four tablets of misoprostol on day two or day three)	591	301 (50.9%)
Incorrect response (any other combination)	591	25 (4.2%)
“Don’t know”	591	265 (44.8%)

The limit of gestation for medical abortion was extended from 7 to 9 weeks by the drug comptroller. Because of the slight ambiguity we allowed any response between 7 and 9 weeks inclusive.

### Knowledge and practice

In Table 5 we examine the practice of pharmacists, comparing this to their knowledge. Only 106 (38.5%) pharmacists asked clients the timing of the last menstrual period and 38 (13.8%) requested to see a doctor’s prescription—a legal requirement in India. Counselling on dosage and direction of use was given to 172 (62.5%) clients, but advice on what to expect, warning signs of possible complications or post-abortion family planning was rarely offered. Only 59 (21.5%) pharmacists correctly advised on the gestational limit for medical abortion, 97 (35.3%) provided correct information on how many and when to take the tablets in a combination pack, and 78 (28.4%) gave accurate advice on where to seek medical attention in case of complications.

**Table 5:** Practice and knowledge (undercover patient and interview data).

	Practice (undercover patient data)		Knowledge (interview data)		P value of difference
	n	Mean	n	Mean	
Asked timing of last menstrual period	275	106 (38.5%)	275	185 (67.3%)	<0.001
Asked to see prescription	275	38 (13.8%)	275	97 (35.3%)	<0.001
Offered any advice on dosage and directions of use	275	172 (62.5%)	275	202 (73.5%)	0.006
Offered any advice on what to expect after taking drug	275	42 (15.3%)	275	35 (12.7%)	0.385
Offered any advice on warning signs of complications	275	13 (4.7%)	275	72 (26.2%)	<0.001
Offered any advice on post abortion family planning	275	4 (1.5%)	275	19 (6.9%)	0.002
Offered any advice on where to seek medical attention	275	4 (1.5%)	275	69 (25.1%)	<0.001
Correctly advised heavy bleeding is a warning sign to seek care*	275	136 (49.5%)	275	250 (90.9%)	<0.001
Correctly advised severe lower abdominal pain is a warning sign to seek care*	275	35 (12.7%)	275	62 (22.5%)	0.003
Correctly advised severe diarrhoea or vomiting is a warning sign to seek care*	275	6 (2.2%)	275	79 (28.7%)	<0.001
Correctly advised high fever is a warning sign to seek care*	275	14 (5.1%)	275	77 (28.0%)	<0.001
Correctly advised shivering is a warning sign to seek care*	275	11 (4.0%)	275	40 (14.5%)	<0.001
Correctly advised on weeks pregnant MA can be used*	275	59 (21.5%)	275	96 (34.9%)	0.0005
Correctly advised on how many and when to take MA pills*	275	97 (35.3%)	275	187 (68.0%)	<0.001
Correctly advised on where to seek care from if needed*	275	78 (28.4%)	275	266 (96.7%)	<0.001

Sample limited to pharmacists who were approached by an undercover patient and offered to sell any drug to induce an abortion. P value is from a Wilcoxon signed rank sum test of the difference between knowledge and practice.

* denotes that the undercover patient prompted for advice related to these indicators if no advice was volunteered by the pharmacist. Correct responses for the following indicators were defined as: 1) Correctly advised on weeks pregnant medical abortion can be used—responses between 7 and 9 weeks inclusive were deemed correct; 2) Correctly advised on how many and when to take medical abortion pills—one tablet of mifepristone on day one, followed by four tablets of misoprostol on day two or day three; and 3) Correctly advised on where to seek care from if needed—any response that includes private doctor, private hospital, primary health centre, district hospital, tertiary hospital or medical college.

The gap between practice and knowledge was generally large (Table 5). For example, two thirds of pharmacists (67.3%) knew to ask clients the timing of the last menstrual period but only 38.5% did so in practice. Pharmacists requested to see a doctor’s prescription in only 13.8% of undercover patient interactions, while 35.3% knew this was a question they should ask. Heavy bleeding was widely recognised (90.9%) as a warning sign but only 49.5% of pharmacists gave advice on this matter. Many pharmacists (68.0%) knew how many and when to take the pills in a combination pack but a minority (35.3%) offered the correct advice in practice. The vast majority of pharmacists (96.7%) knew where to seek care if a complication arose, while only 28.4% gave such advice.

## Discussion

The private retail market for medical abortion in Madhya Pradesh is extensive, but the quality of advice given to clients is poor. There were on average 13 pharmacies per retail market, over two-thirds of whom offered medical abortion. Local market availability—a better indicator of whether local consumers have access to medical abortion—was almost universal in urban settings, albeit lower in rural areas. Knowledge on the legality of abortion was poor, and very few chemists asked to see a prescription. Only a third of pharmacists asked the gestational age of the woman or correctly advised on how many and when to take the drugs. Advice on post-abortion family planning was almost nonexistent.

The extensive nature of private healthcare provision in India is well documented.[20] However, much less is known about the scale of the medical abortion market.[21, 22] One study in Bihar and Jarkhand examined different types of abortion drugs available but only in pharmacists reporting to stock or sell an abortion drug.[23] Our study suggests that medical abortion is nearly universally available in urban settings, and in half of retail markets in rural areas. Whether the latter reflects *access* in rural areas is arguable since we excluded smaller villages where very few pharmacies are thought to operate.

Why is the market for medical abortion so seemingly vibrant? On the supply side, regulation of pharmacists and medical abortion is weakly enforced, as suggested by the fact that few pharmacists requested to see a prescription. On the demand side, a high proportion of women want an abortion by the time they reach thirty,[14] there is a strong preference for aborting at home,[8, 24] and the cost of medical abortion is far lower than surgical procedures.[13] The relationship between contraception and abortion is difficult to unpick.[25, 26] But theory suggests that as desired family size declines, unwanted fertility will rise if the means to prevent pregnancies are not available or taken up.[27] In this sense, the market for medical abortion is responding to the considerable numbers of unintended pregnancies.[28] While the ideal number of children in Madhya Pradesh has fallen, there has been little change in the use of spacing methods of contraception and the level of use remains extremely low at 9.6%.[17] Reliance on abortion will only decrease when women are able to control their fertility through other means, particularly temporary methods of contraception. Over the last twenty years, the Indian Government has tried to move towards a system that addresses individual’s needs but progress has been limited and uneven, partly because some service providers are reluctant to adopt the principle of informed choice.[29]

The implications of the expansion of the retail market for medical abortion for women’s health are not entirely known. It is encouraging that the majority of the products on the market were combination packs which may help women to take the correct dosage. However, the poor quality of advice given by pharmacists is worrying, albeit consistent with a number of studies in India and elsewhere.[22, 30–36] Since few pharmacists established the woman’s gestational age or gave the correct advice on how many and when to take the drugs, it is possible that a sizeable number of women who source the combination pack drugs from pharmacists directly take them incorrectly or too late in pregnancy. Health providers we spoke to in hospitals anecdotally report a fall in severe abortion complications coupled with a rise in the number of women admitted for prolonged bleeding, some of whom develop severe anaemia. There are very few data on the incidence of abortion morbidity in India.[37] The WHO multi country survey on maternal near miss found very few cases due to abortion in hospitals in India but case definitions were very stringent and cases may have been missed because abortion was not the focus of the study.[38] The contribution of medical abortion to women’s health in India thus warrants further investigation.

Our study had several limitations. First, there was no sampling frame with which to select pharmacies and we sampled proportional to population size within urban and rural districts. The characteristics of pharmacists and medical abortion availability did not vary by stratum, nor was there any indication that knowledge or practice varies, and we are confident that the findings represent the situation on the ground. Second, we only interviewed one person behind the counter and when comparing practice with knowledge there is no guarantee that we captured data on the same person. Reassuringly, 99% of respondents in interview were responsible for the daily running of the shop. Third, we are unable to rely on the interview data to measure availability of medical abortion and we place much greater weight on the data obtained through the use of undercover patients. Finally, our study was limited to the retail sector and it would be informative to know more about medical abortion provision in the public sector.

The widespread informal use of medical abortion represents challenges for the definition and measurement of unsafe abortion. The WHO defines unsafe abortion as a procedure for terminating a pregnancy performed by persons lacking the necessary skills or in an environment not in conformity with minimal medical standards, or both.[39] While pharmacists offer women an evidence-based procedure, the advice they offer does not conform to minimal medical standards so the abortion is, strictly speaking, unsafe. Yet most observers will agree that insofar medical abortion has displaced clandestine or riskier methods such as traditional means of home abortion, fewer women will suffer severe complications or death. The WHO has recently advocated for a more multi-dimensional assessment of the safety of induced abortion, including the measurement of morbidity.[39] There is a dearth of data on the incidence of abortion complications and definitions vary substantially.[37] Much can be learned from the field of obstetrics, where standard definitions are available for complications such as severe haemorrhage, septicaemia, and anaemia, including near miss.[37, 38] Applying this knowledge to gain a better understanding of the health consequences of the extensive medical abortion market should be a priority.

Pharmacists appear to be liberal in selling medical abortion without prescription yet provide poor counselling to customers, in a context where there is little privacy and interactions between the pharmacist and the patient are short. The growth in the unregulated retail market for medical abortion poses a challenge for government and other agencies concerned with women’s reproductive health. There are some examples of successful efforts to improve practice of private pharmacists, through training and other means.[40–42] But the substantial knowledge-practice gap observed here suggests that increasing knowledge through training alone may not be sufficient to change practice. Ultimately, efforts to improve practice will require greater understanding of the incentives faced by pharmacists and how these influence their practice. In the present situation, clear information leaflets in local languages and stricter enforcement of existing government regulations without harming access may be the most feasible path to improvement.

## Conclusions

The retail market for medical abortion is extensive, but the quality of advice given to patients is poor. Although the contribution of medical abortion to women’s health in India is poorly understood, there is an urgent need to improve the practices of pharmacists selling medical abortion.

## Supporting Information

S1 Table Characteristics of selected districts. Data are from the Annual Health Survey 2011, and Census 2011. Values in brackets are for Guna district taken from the Annual Health Survey 2011. Ashok Nagar used to be part of Guna district.
(DOCX)

S2 Table Characteristics of chemists. (DOCX)

S3 Table Characteristics of chemists interviewed and visited by undercover patients. (DOCX)

S4 Table Knowledge of abortion amongst interviewed sample and undercover patient sample. (DOCX)

S5 Table Additional knowledge indicators based on vignette of a medical abortion customer. (DOCX)

S1 Fig Brands of medical abortion combination pack available in the market using undercover patient and interview data. Brands available from the interview data refer to brands of medical abortion combination pack currently in stock. In the legend the number of local markets is shown in parentheses.
(TIF)

S1 Appendix (DOCX)
