# Supplementary material for: Delivering Medical Abortion at Scale: A Study of the Retail Market for Medical Abortion in Madhya Pradesh, India
Source: PLoS One. 2015 Mar 30;10(3):e0120637. doi: 10.1371/journal.pone.0120637 (PMC4379109; doi:10.1371/journal.pone.0120637)
Supplement: S1 Table — Data are from the Annual Health Survey 2011, and Census 2011. Values in brackets are for Guna district taken from the Annual Health Survey 2011. Ashok Nagar used to be part of Guna district. (DOCX) [file pone.0120637.s001.docx]

| District | Population | Urban population | Population density | Sex ratio at birth | Crude birth rate | Abortion rate | Literacy |
| --- | --- | --- | --- | --- | --- | --- | --- |
| Bhopal | 2.37m | 81% | 854 | 912 | 19.2 | 5.4% | 88% |
| Indore | 3.27m | 74% | 839 | 862 | 19.6 | 9.9% | 87% |
| Gwalior | 2.03m | 63% | 445 | 807 | 18.5 | 7.0% | 84% |
| Satna | 2.23m | 30% | 297 | 929 | 28.8 | 3.5% | 73% |
| Ashok Nagar | 0.84m | 17% | 181 | (900) | (28.7) | (1.0%) | 69% |
| Umaria | 0.64m | 14% | 158 | 946 | 29.9 | 3.0% | 70% |
