# Supplementary material for: Delivering Medical Abortion at Scale: A Study of the Retail Market for Medical Abortion in Madhya Pradesh, India
Source: PLoS One. 2015 Mar 30;10(3):e0120637. doi: 10.1371/journal.pone.0120637 (PMC4379109; doi:10.1371/journal.pone.0120637)
Supplement: S2 Table — (DOCX) [file pone.0120637.s002.docx]

|  | Total  (n=591) | City  (n=283) | Town (block)  (n=265) | Rural (village)  (n=43) |
| --- | --- | --- | --- | --- |
| Respondent is owner | 394 (66.7%) | 178 (62.9%) | 192 (72.5%) | 24 (55.8%) |
| Responsible for daily running of shop | 585 (99.0%) | 279 (98.6%) | 263 (99.2%) | 43 (100%) |
| Hindu | 530 (89.7%) | 245 (86.6%) | 247 (93.2%) | 38 (88.4%) |
| Muslim | 35 (5.9%) | 27 (9.5%) | 7 (2.6%) | 1 (2.3%) |
| Christian | 2 (0.3%) | 2 (0.7%) | 0 (0.0%) | 0 (0.0%) |
| Sikh | 6 (1.0%) | 6 (2.1%) | 0 (0.0%) | 0 (0.0%) |
| Other religion | 17 (2.9%) | 3 (1.1%) | 11 (4.2%) | 3 (7.0%) |
| No religion | 1 (0.2%) | 0 (0.0%) | 0 (0.0%) | 1 (2.3%) |
| Primary education | 4 (0.7%) | 1 (0.4%) | 3 (1.1%) | 0 (0.0%) |
| Secondary education | 19 (3.2%) | 11 (3.9%) | 8 (3.0%) | 0 (0.0%) |
| Higher education (12 years or more) | 568 (96.1%) | 271 (95.8%) | 254 (95.8%) | 43 (100.0%) |
| People working in chemist (persons) | 2.3 (1.2) | 2.6 (1.4) | 2.0 (0.9) | 1.7 (0.6) |
| Any staff with masters in pharmacy | 21 (3.6%) | 17 (6.0%) | 4 (1.5%) | 0 (0.0%) |
| Any staff with bachelors in pharmacy | 175 (29.6%) | 95 (33.6%) | 69 (26.0%) | 11 (25.6%) |
| Any staff with diploma in pharmacy | 248 (42.0%) | 118 (41.7%) | 115 (43.4%) | 15 (34.9%) |
| Any staff with certificate course in pharmacy | 45 (7.6%) | 18 (6.4%) | 25 (9.4%) | 2 (4.7%) |
| Any staff with medical degree | 15 (2.5%) | 7 (2.5%) | 8 (3.0%) | 0 (0.0%) |
| No staff with any health qualification | 109 (18.4%) | 45 (15.9%) | 51 (19.2%) | 13 (30.2%) |
| Open per week (days) | 6.6 (0.6) | 6.5 (0.5) | 6.7 (0.6) | 6.9 (0.3) |
| Number of years established (years) | 12.7 (11.3) | 12.4 (10.0) | 13.6 (12.8) | 9.4 (9.8) |
| Drugs stored in shop | 579 (98.0%) | 279 (98.6%) | 257 (97.0%) | 43 (100.0%) |
| Business owns car | 40 (6.8%) | 16 (5.7%) | 19 (7.2%) | 5 (11.6%) |
| Business owns motorbike | 502 (84.9%) | 272 (96.1%) | 202 (76.2%) | 28 (65.1%) |
| Know any doctor locally | 510 (86.3%) | 240 (84.8%) | 230 (86.8%) | 40 (93.0%) |
| Average customers per day | 56.3 (63.6) | 70.5 (83.1) | 44.0 (32.2) | 38.4 (36.7) |
|  |  |  |  |  |
