# Supplementary material for: Delivering Medical Abortion at Scale: A Study of the Retail Market for Medical Abortion in Madhya Pradesh, India
Source: PLoS One. 2015 Mar 30;10(3):e0120637. doi: 10.1371/journal.pone.0120637 (PMC4379109; doi:10.1371/journal.pone.0120637)
Supplement: S3 Table — (DOCX) [file pone.0120637.s003.docx]

|  | Interview sample (n=591) | Undercover patient sample (n=359) |
| --- | --- | --- |
| Respondent is owner | 394 (66.7%) | 241 (67.1%) |
| Responsible for daily running of shop | 585 (99.0%) | 356 (99.2%) |
| Hindu | 530 (89.7%) | 329 (91.6%) |
| Muslim | 35 (5.9%) | 15 (4.2%) |
| Christian | 2 (0.3%) | 1 (0.3%) |
| Sikh | 6 (1.0%) | 1 (0.3%) |
| Other religion | 17 (2.9%) | 12 (3.3%) |
| No religion | 1 (0.2%) | 1 (0.3%) |
| Primary education | 4 (0.7%) | 3 (0.8%) |
| Secondary education | 19 (3.2%) | 8 (2.2%) |
| Higher education (12 years or more) | 568 (96.1%) | 348 (96.9%) |
| People working in chemist (persons) | 2.3 (1.2) | 2.3 (1.2) |
| Any staff with masters in pharmacy | 21 (3.6%) | 12 (3.3%) |
| Any staff with bachelors in pharmacy | 175 (29.6%) | 110 (30.6%) |
| Any staff with diploma in pharmacy | 248 (42.0%) | 151 (42.1%) |
| Any staff with certificate course in pharmacy | 45 (7.6%) | 23 (6.4%) |
| Any staff with medical degree | 15 (2.5%) | 9 (2.5%) |
| No staff with any health qualification | 109 (18.4%) | 67 (18.7%) |
| Open per week (days) | 6.6 (0.6) | 6.6 (0.5) |
| Number of years established (years) | 12.7 (11.3) | 13.2 (11.4) |
| Drugs stored in shop | 579 (98.0%) | 349 (97.2%) |
| Business owns car | 40 (6.8%) | 27 (7.5%) |
| Business owns motorbike | 502 (84.9%) | 301 (83.8%) |
| Know any doctor locally | 510 (86.3%) | 316 (88.0%) |
| Average customers per day | 56.3 (63.6) | 62.8 (76.4) |
|  | | |
