# Supplementary material for: Delivering Medical Abortion at Scale: A Study of the Retail Market for Medical Abortion in Madhya Pradesh, India
Source: PLoS One. 2015 Mar 30;10(3):e0120637. doi: 10.1371/journal.pone.0120637 (PMC4379109; doi:10.1371/journal.pone.0120637)
Supplement: S4 Table — (DOCX) [file pone.0120637.s004.docx]

|  | Sample interviewed  (n=591) | Sample visited by undercover patients  (n=359) |
| --- | --- | --- |
| **Is abortion legal in India?** | | |
| Correct response (yes) | 167 (28.3%) | 112 (31.2%) |
| Incorrect response (no) | 405 (68.5%) | 239 (66.6%) |
| “Don’t know” | 19 (3.2%) | 8 (2.2%) |
| **How do you calculate gestation age?** | | |
| Correct response (beginning of last menstrual period) | 347 (58.7%) | 236 (65.7%) |
| Incorrect response (any other response) | 45 (7.6%) | 22 (6.1%) |
| “Don’t know” | 199 (33.7%) | 101 (28.1%) |
| **Medical abortion is permissible up to how many weeks pregnant?** | | |
| Correct response (between 7 and 9 weeks inclusive) | 183 (31.0%) | 128 (35.7%) |
| Incorrect (more than 9 weeks) | 164 (27.7%) | 96 (26.7%) |
| Incorrect (less than 7 weeks) | 163 (27.6%) | 104 (29.0%) |
| “Don’t know” | 81 (13.7%) | 31 (8.6%) |
| **How should combination pack medical abortion drugs be administered (number of pills and timing)?** | | |
| Correct response (one tablet of mifepristone on day one, followed by four tablets of misoprostol on day two or day three) | 301 (50.9%) | 228 (63.5%) |
| Incorrect response (any other combination) | 25 (4.2%) | 16 (4.2%) |
| “Don’t know” | 265 (44.8%) | 115 (32.0%) |
