# Supplementary material for: Delivering Medical Abortion at Scale: A Study of the Retail Market for Medical Abortion in Madhya Pradesh, India
Source: PLoS One. 2015 Mar 30;10(3):e0120637. doi: 10.1371/journal.pone.0120637 (PMC4379109; doi:10.1371/journal.pone.0120637)
Supplement: S5 Table — (DOCX) [file pone.0120637.s005.docx]

|  | Sample interviewed  (n=591) | Sample visited by undercover patients  (n=359) |
| --- | --- | --- |
| **Before selling medical abortion kit, what questions would you ask the client (multiple responses)?** | | |
| Enquire about gestational age | 234 (39.6%) | 169 (47.1%) |
| Request to see prescription | 178 (30.1%) | 123 (34.3%) |
| Enquire whether doctor has been seen | 110 (18.6%) | 74 (20.6%) |
| Suggest go to see a doctor | 290 (49.1%) | 168 (46.8%) |
| **Before selling the medical abortion kit, what pieces of advice would you give the client (multiple responses)?** | | |
| Dosage | 261 (44.2%) | 188 (52.4%) |
| When to take tablets | 228 (38.6%) | 154 (42.9%) |
| What to expect after taking drug | 52 (8.8%) | 45 (12.5%) |
| Potential side effects | 129 (21.8%) | 85 (23.7%) |
| Post abortion family planning | 40 (6.8%) | 23 (6.4%) |
| Where to seek medical care | 145 (24.5%) | 89 (24.8%) |
| **What are the warning signs of side-effects or complications from medical abortion drugs (multiple responses)?** | | |
| Heavy bleeding | 493 (83.4%) | 318 (88.6%) |
| Severe lower abdominal pain | 123 (20.8%) | 83 (23.1%) |
| Severe diarrhoea or vomiting | 140 (23.7%) | 98 (27.3%) |
| High fever | 126 (21.3%) | 94 (26.2%) |
| Shivering | 79 (13.4%) | 43 (14.8%) |
|  | | |
