# Supplementary material for: Delivering Medical Abortion at Scale: A Study of the Retail Market for Medical Abortion in Madhya Pradesh, India
Source: PLoS One. 2015 Mar 30;10(3):e0120637. doi: 10.1371/journal.pone.0120637 (PMC4379109; doi:10.1371/journal.pone.0120637)
Supplement: S1 Appendix — (DOCX) [file pone.0120637.s007.docx]

**Appendix**

Delivering medical abortion at scale: a study of the market for medical abortion in Madhya Pradesh, India

**Inclusion Criteria and Sample Size Calculations**

**Ensuring Participation**

**Ethical Considerations**

# Inclusion Criteria and Sample Size Calculations

Within each sampled cluster, a census of health providers was done to collect basic information on the location of each provider and their type. In this listing of health providers we included all types of government health facility, all types of private health facility in the for-profit and not-for-profit sector, and pharmacies. We excluded homeopathic facilities, ayurvedic facilities, community health workers, and shops (selling drugs). Pharmacies were defined as any outlet whose primary business was selling medicines, irrespective of whether they were appropriately trained as required by law. The information from the listing exercise provided the sample frame with which to then randomly select pharmacists for interview.

The sample size of pharmacies was driven largely by budget considerations and feasibility. As stated in the study protocol, in comparing the availability of medical abortion drugs between urban and rural areas, a sample of 450 pharmacies with 80% power, an alpha of 0.05, and an intraclass correlation of 0.2 would be sufficient to detect the difference between 20% in rural areas and 35% in urban (city and town combined) areas.

# Ensuring Participation

When designing the study an important concern was that pharmacists would not be willing to participate in a study on medical abortion. Hence we designed the questionnaire and trained the field researchers carefully to ensure we achieved as high a response rate as possible. Our strategy to maximise response rate included: 1) introducing the study to participants as a study about reproductive health; 2) asking questions about the characteristics of the pharmacists first so as to ease them into the interview; 3) asking questions on the availability of antibiotics (for which there is no stigma or controversy) before asking about availability of medical abortion drugs; and 4) placing the questions on knowledge of medical abortion towards the end of the questionnaire. We believe this strategy helped us achieve a relatively high response rate of 87 percent.

# Ethical Considerations

The study protocol outlined the main ethical issues. Careful attention was be given to the ethics of implementing the undercover patient component of the data collection. It is worth noting that a large study using standardised patients in Madhya Pradesh was recently conducted successfully having gained ethical approval (Das et al 2012).

As stated in the study protocol, the following ethical issues were given consideration. First, the study must pose no major risk to the undercover patients themselves. This was deemed unlikely to be the case since the interaction between patient and pharmacist would require no invasive procedures or examinations. Second, the undercover patients must be credible which means they will be unknown to the pharmacists. A deception design in which the pharmacists are not informed of the impending visit by an undercover patient was requested on the basis that the risks to pharmacists and undercover patients are minimal. At the same, data on the actual practices of pharmacists are very limited and the value of the research is therefore high. The undercover patients did pay for non-medical products offered (eg. homeopathic drugs) but did not purchase medical abortion drugs. Undercover patients were intensively trained and given considerable support when out in the field. A screening questionnaire was used to ensure the selection process recruited suitable undercover patients. A training manual was developed to guide the training of the undercover patients.

Informed consent form was read and given to pharmacists before interview on the purpose of the study, the broad content of the interview, the confidentiality of the data, the voluntary nature of the interview, the possibility of stopping the interview at any time without reason, and the contact details of the principal investigators. Written consent was sought from pharmacists and needed to be given prior to the interview commencing. Written consent was also be sought from potential undercover patients prior to recruitment since they were participants in the study.

Other more general ethical issues to address included: processes to maintain confidentiality of the data collected (ie. secure storage of the questionnaires; anonymised data when digitised; secure storage of data including encryption), training of interviewers in value clarification so that they approach the issue of abortion with great sensitivity, how to handle breaches of confidentiality by members of the data collection and research teams, and appropriate dissemination of data.

An important ethical risk of the study pertained to breaches of confidentiality. The study made every effort to minimise the risk of breaches of confidentiality, particularly in relation to data management and the linking of datasets. A number of steps were be taken:

1. Data on pharmacists and undercover patients were entered separately by separate sets of data entry operators in New Delhi, and were linked only by the principal investigators at the time of analysis. In this way, misuse or breach of confidentiality even at the data entry level was reduced by a great extent.
2. Paper questionnaires were stored in a locked cupboard in the Council office and will be destroyed after the data are analysed and results reported.
3. Merged datasets are kept securely and are only accessed by the principal investigators. No other persons have access to the securely kept data.
4. The datasets contain no information that would allow identification of any individual pharmacist or undercover patient. They contain no information on names (of pharmacist, undercover patient and village) or GPS information. The village cluster was be coded such that it is impossible to know the name of the village from the datasets.
5. GPS information was collected using a separate data collection form, entered into digital format separately and the digital format of the data was be stored in its own data file. The pharmacist and undercover patient datasets were not linked to the GPS information dataset, except by the principal investigators at the time of analysis. The geographical location of an individual pharmacist is not identifiable to anyone other than the principal investigators.
6. Data is to be reported in aggregate format only (eg. means, subgroup means, percentages, regression outputs) such that no individual pharmacist can be identified. All these measures are standard procedures employed in surveys (eg. Demographic and Health Surveys).
